# The clinic as a good corporate neighbor

**DOI:** 10.3325/cmj.2013.54.78

**Published:** 2013-02

**Authors:** Hans-Martin Sass

**Affiliations:** Potomac Institute for Policy Studies, Arlington, VA, USA

## Abstract

Clinics today specialize in health repair services similar to car repair shops; procedures and prices are standardized, regulated, and inflexibly uniform. Clinics of the future have to become Health Care Centers in order to be more respected and more effective corporate neighbors in offering outreach services in health education and preventive health care. The traditional concept of care for health is much broader than repair management and includes the promotion of lay health competence and responsibility in healthy social and natural environments. The corporate profile and ethics of the clinic as a good and competitive local neighbor will have to focus on [a] better personalized care, [b] education and services in preventive care, [c] direct or web-based information and advice for general, seasonal, or age related health risks, and on developing and improving trustworthy character traits of the clinic as a corporate person and a good neighbor.

In Memoriam Ivan Šegota

## From health repair to health care

Clinics are not in good shape today in most places, neither financially nor conceptually (1,2). They are squeezed by bureaucratic regulations, long working hours and shifts for staff, financial restrictions, and the lack of incentives. So called quality norms require that individual patients be treated universally according to disease classification norms. Inflexible generic reimbursement schemes keep clinics barely afloat. Bureaucratic regulation of medicinal drugs approves only “one size fits all” remedies not recognizing, eg, well documented individual differences in p-450 cytochrome drug metabolism (3). These inflexible bureaucratic generalizations originally might have been well intended to protect citizens and patients, but they have become obsolete in the 21st century, as they do not take into account the traditional and necessarily future role of a partnership-in-trust between providers and recipients of health care. Compassion and care often are low, staff is burned-out, and there are not many incentives or even opportunities to change things for the better. Clinical routine for providers and recipients is dictated by numbers; but people are neither numbers nor cases, they are individuals as we are, different in age and gender, hopes, angst, expectations, and competencies.

The World Health Organization (WHO) defines health as something static, “a state of complete physical, mental and social wellbeing and not merely the absence of disease and infirmity.” But, health is never just a state, like the state of a rock, a machine, or an automobile; health always is “a process and a balanced result of health-literate and health-competent care of one’s own physical, emotional, and social wellbeing, achieved by competent understanding, modification and enhancement of individual genetic, social, and environmental risk factors and challenges, with the support of health care professionals and through equal and fair access to health care service, including health education and preventive services.” Such a traditional and integrative concept of health has not been implemented in the health repair businesses and services of the 21st century and it is questionable if further narrow bureaucratic regulation will ever get us back to a more integrated model of caring for health in a true partnership among citizens and patients on one side and health care experts of various kind on the other (4).

Two thousand years ago, Galenus of Bergama in Izmir Province, a successful scientist and physician to the Roman elite and to Emperor Marc Aurel, taught his fellow doctors “*non homo universalis curatur, set unus, quique, noster*:” “we don’t cure a universal human being, but an individual one, a special one, ours.” Modern regulations have no room for personalized care (5-7); in shift-based hospital service the development of a personal care between provider and recipient is nearly impossible. How do we get back to personalized care, beyond the static rules and regulations? Sun Simiao, called the “King of Medicine” since the Tang Dynasty in the 6th century, differentiated in his famous book “Essential Formulas for Emergencies Worth a 1000 Pieces of Gold” between three types of doctors: “A superior doctor takes care of the state, an average doctor takes care of the person, an inferior doctor takes care of the disease.” (8). Do we only have inferior doctors today, just taking care of diseases and symptoms? The excellent success of modern medicine based exclusively on natural science and great breakthroughs in hygiene, anesthesia, antibiotics, and medical machinery definitely has contributed to the deformation of traditional integrative medicine. But in our pursuit of repair competence, our experts and clinics and we ourselves do not have much time for compassion. Also, we lost the broader picture of “public health” or integrative health policy and culture: healthy lifestyles, healthy families and healthy neighborhoods, and healthy natural and social environment for the 21st century.

How do we recover the treasures of the past in winning the future? Will citizens and patients or regulators and politicians or insurers lead the way out of the dead-ends of norms and regulations? My recommendation is that clinics try to take the first steps themselves to develop step by step heath repair shops to truly health care centers. In doing so, they will become better corporate citizens, improve they neighborhood and their own recognition as a trustworthy partners in all fields of caring for the health of all individuals, all ages, all natural and social environments. A walk of 1000 miles begins with a first few steps, says an old proverb. Not unrelated to the success of modern medical science, the knowledge gap between the lay and the expert and among experts of various kind is much larger than it had been over the centuries. I will suggest a few preliminary steps, which can be taken by clinics in the change for healthy health care. There are outreach programs in health information and preventive health care; there is the integration of ambulatory and stationary services in “televisits;” there is the option for personalized health care such as in old-age care and personalized drug treatment. These additional services will need to be funded by additional and non-bureaucratic sources but they will improve the recognition of the clinic as a good and trustworthy neighbor. The clinic is the main authority in rural areas far beyond the city limits; in urban centers there might be a competition between clinics. Thus the character profile of good clinics as a trustworthy corporate neighbor becomes most important.

## Re-appreciating classical health care wisdom

The Suwen classical medical Chinese text, attributed to the Yellow Emperor 4500 years ago and comparable in influence only to the Hippocratic tradition in Europe, reminds already the people of those days: “The sages did not wait until the sickness is there to cure the sickness, they cure it before it takes place ... if one only waits until the sickness is there and then uses medicine to cure it, that is not different from waiting until one is thirsty and then starting to dig a well.” (8). What can modern clinics learn from the Yellow Emperor? They can become good corporate citizens helping their neighbors and the community in sharing their expertise in keeping and improving health by lifestyle, nutrition, physical exercise, stress management, and the development of age-related health care cultures. In the Hippocratic tradition dietetics Galen summarizes six remedies for integrated health care: light and air (*aer*), eating and drinking (*cibus et potus*), work and rest (*motus et quies*), sleep and wake (*somnus et vigilia*), secretion and excretion (*secreta et excreta*), and stimulation of the mind (*affectus animi*). Which doctor and which clinic do you know, who are following these well proven millennia old rules? And how would they get paid for doing so? Enlighted German doctor and pharmacist Friedrich Hoffmann (1660-1742), whose Dr Hoffmann drops are still available today in German apothecaries, summarized the six dietetic rules, but added a seventh caveat: “1. Stay away from everything which is unnatural. – 2. Be careful with changes as routine often becomes our 2nd nature. – 3. Be happy and balanced, that is the best remedy. – 4. Stay in clean air and moderate temperature as much as possible. – 5. Buy the best nutrition which goes easily in and out of the body. – 6. Choose foods according to your bodily activity and relaxation. – 7. When you love to be healthy, run away from physicians and from all drugs.” Dr Hoffmann of Halle an der Saale warned about the quacks of his time, but would he have been wrong about modern doctors following WHO and professional classification and reimbursement schemes and as repair shops with meager quality-of-life for customers and providers? What can be done to build and to improve health literacy and health care competence among lay people, doctors, and nurses alike, and who could or should do it?

Full scale centers for integrated health care: Repair shops must build outreach instruments to become true and successful neighbors trusted for their expertise and advice on all health care matters. These are: 1) a printed monthly journal to friends of the clinic, 2) a regular online information bulletin informing and advising on timely issues such as protection against sunburn in the summer and cold and flu in the winter, also routine issues such as diabetes, nutrition, physical exercise, age related adjustment and appreciation of lifestyle, 3) classes offered for seasonal or specialized health care issues (those are mandated by Chinese hospitals in the service to their respective community, thus establishing clinics as competent and compassionate neighbor), 4) a homepage organized alike the *www.realage.com* or *www.netdoctor.com.uk* with a full information and advice service, including a self-test program for healthy and sick lay people; such a Web site would extend the good neighbor service potentially on a global scale.

## Integrating ambulatory and stationary care

The lack of integration between ambulatory services provided by doctors outside of clinics and in-house services has been a major shortcoming of modern repair care systems. Quite a number of proposals have been made to introduce and to improve an integrated service, which can be based on the use of safe and efficient modern communication technology (5). This might actually lead also to better service for rural populations when “televisits” by highly experienced experts will be possible with the help of local nurse practitioners or other less specialized personnel. Modern technology also allows for direct alerting, monitoring, informing, and advising of patients in regard to taking medicines, controlling and reporting blood pressure and other vital signs.

## Personalized genetic and age-related information in health care

We all have different genetic traits which make us susceptible to certain diseases and/or the protection against others. The acute phase of late onset genetic disorders such as ADPKD can be postponed, while others such as various forms diabetes can be batter managed by informed and well guided carriers. Hospitals more and more can specialize in outreach programs for one or the other of these genetic variations and make a name for themselves in the national and even global community. Modern pharmacogenetics provide knowledge about individual liver enzyme properties of the p-450 cytochrome in nutrition and in particular drug metabolism. National and international oversight bodies for licensing and approving drugs have failed to issue license and to guide physicians and consumers based on these individual properties (3). For example, the metabolism of diazepam, eg, is quite different in Han people than in Caucasian; Asian countries need to evaluate efficiency and dosage of drugs developed and approved for countries with Caucasian majority; but different metabolizing types are evident not only between, but also within all genetic groups. Similar information should be requested for the use in special populations such as the elderly or minority groups. Since governmental authorities unfortunately have not yet provided specific licensing for specific metabolizers, Clinics along with professional medical associations are the only ones who can and should request research data from drug companies stating that otherwise these medications would not be prescribed. This would be the competent and compassionate service of a good corporate neighbor.

## The character profile of a good corporate neighbor

What makes the character of a good clinic as a competent and compassionate neighbor and what do neighbors expect from a good clinic? We find the answer in Confucian doctor’s Yang Chuan (300AD) advice to the lay community: “Trust only those physicians who have the heart of humaneness and compassion, who are clever and wise, sincere and honest” (8). We do not have to reinvent the wheel and simply can translate Dr Yang Chuan’s suggestion into the 21st century of corporate medicine: “Trust only those hospitals who have the heart of humanness and compassion, who are clever and wise, sincere and honest.” Hospital ethics like many other fields of corporate ethics is based on partnership-in-trust. Some time ago in this journal (9), I had suggested moral maxims for stakeholders in health care: for politicians and regulators as the ones who set the framework within which individuals, corporations, and insurers have to work, lay people as the main and central agents, health care professionals as the experts, and insurers as the financial facilitators. However, even more important is the translation of character traits of personally and professionally successful, compassionate and respected persons into the world of corporate persons. This is an issue for translational ethics. Over millions of years and in all cultures and traditions we can find the same properties and gifts which have made us humans distinct from other forms of life and which had been instrumental in developing the cultures of previous and contemporary social and natural environments in building houses and machinery, infrastructure, corporations, and networks of all kind: our capacities for communication and cooperation, for compassion and for developing competence and expertise. The following list does not need much explanation and has been used successfully in clinical service and ethics training in association with case studies for their concretization (Table 1) (10).

**Table 1:** The basics for personalized medicine and clinics as good corporate neighbors

## A global positioning system (GPS) for healthy health care

To develop a future oriented global positioning system for the clinic as a successful corporate person, we just have to look into old and new traditions in leadership and in developing and protecting cultures and communities. Fritz Jahr, who coined the term “bioethics” in 1927, made an exemplary good use of classical moral teaching by interpreting the Fifth Commandment “Thou shall not kill” in 1934. He described it very bluntly in three steps: 1) do not kill yourself by not living a healthy lifestyle; 2) do not kill the community by not providing public health; 3) do not kill healthy and integrated living environments in not respecting all forms of life in adhering to the Bioethical Imperative “Respect every living being in principle as an end in itself and treat it, if possible, as such” (11).

Close to 2500 years earlier the Tao had pointed out that healthy communities are based on the integrated virtues of people, families, neighborhoods, states, and the global community: “Cultivate yourself and virtue becomes true; cultivate the family and virtue will be full; cultivate the village, and virtue will grow; cultivate the country, and virtue will be rich; cultivate the world, and virtue will be wide” [No. 54]. We may translate Lao Tze’s 2500 years old wisdom into an Bioethics Imperative for the 21th Century: “Cultivate yourself and life and virtue becomes true; cultivate social and natural environments and virtue will be full; cultivate communication and cooperation, and life will grow; cultivate compassion and competence, and life will be rich; cultivate life, and world and virtue will be wide.” As a Clinical Ethics Imperative for the 21st Century such a maxim would read: “Cultivate the Clinic and virtue becomes true; cultivate your neighborhood and virtue and health will be full; cultivate communication and cooperation, and healthcare will grow; cultivate compassion and competence, and health care will be rich; cultivate global health, and health and virtue will be wide.”

## 

**Table:** 

	Lay person	Expert person	Corporate person
Communication	Educate yourself in health care matters, read books and get expert advice	Communicate with and advice your patient in easy to understand language, follow up on previous advice and patient’s questions	Provide for open communication inside and outside
Cooperation	cooperate with experts in prevention, diagnosis and therapy, follow advice and be compliant	diagnose and integrate the medical and value status of your patient, support lay health competence	provide translational service between experts in medicine, care and leadership, support open communication and cooperation with patients and the community
Competence	develop your health literacy and responsibility, use the internet and personal expert advice to improve lifestyle and health	be the best possible expert and continuously educate yourself in improving your technical and personal skills, educate your patients	provide for a climate of compassionate competence on all levels, manage your institution for optimum success in business and service
Compassion	be fair and understanding in communication and cooperation with others, expect and give dignified cooperation and reciprocity in understanding human failures and limitations	provide your professional service in a civilized and compassionate manner; treat fellow experts and patients as fellow humans not just as stakeholders	make competent compassion an overriding principle in your corporate strategy and corporate ethics; provide for continuous training and review of compassionate and humane service
Cultivation	improve and cultivate your health competence; develop and cultivate a healthy and happy lifestyle; cultivate the care of your physical and mental limitations	learn from failure and carelessness, from professional incompetence and lack of cooperation; continuously improve procedures and team work; use informed contracts rather than informed consent for stakeholder reciprocity	make improvement in professionalism and teamwork a high priority; develop and display a trustworthy corporate profile as leadership in internal and public relations; support the replacement of informed consent models by those of informed contract
